# Book Review: The Will to Meaning: Foundations and Applications of Logotherapy

**DOI:** 10.3389/fpsyg.2017.02106

**Published:** 2017-12-06

**Authors:** Khushali Adhiya-Shah

**Affiliations:** Department of Psychology, Mithibai College of Arts, Mumbai, India

**Keywords:** Viktor Frankl, the will to meaning, logotherapy, noogenic neurosis, meaning in life

Succeeding his book *Man's Search for Meaning*, Viktor Frankl releases an ideal issue of this book, *The Will to Meaning*. While the former book aimed at introducing the idea of saying yes to life in spite of everything (including suffering) and finding meaning, *The Will to Meaning* precisely explains its procedure, technical aspects and working mechanisms.

Written by Austrian neurologist–psychiatrist and a Holocaust survivor Victor Frankl, this book is profoundly deep. Frankl (1988), regarded as the father of Logotherapy, has been well-recognized for his ideas on finding meaning in life, application of his techniques-paradoxical intention, depersonalization and Socratic dialogue in psychotherapy, diagnosing the current crisis of Sunday boredom, and existential vacuum in the general population. His school of thought invites meaningful treatment of phobias, pain and guilt, despair and grief.

Frankl (1988) seems to have addressed the many queries raised by a clinician (after the first book) sufficiently well in this second book. Throughout this book, Frankl reports the careful analysis done by him of what can be and what definitely cannot be meant by “will to meaning.” He uses a simple language to explain his powerful ideas, supplemented by his already established ways of using personal experiences to explain concepts further. Additionally, an interesting aspect of this book includes his creativity in using geometrical patterns to explain the view of human nature. In the end, Frankl also encourages such creativity in those unique individuals who wish to take Logotherapy ahead. The author also uses a strong, assertive language to express the differences between Logotherapy and the classical theories of Freud and Alder. And simultaneously, he also respectfully regards their theories as the “giants on the shoulders of which the dwarf” (Logotherapy) can see further ahead. One can observe a conscious effort in the choice of the words used by him and the connotation that they imply from his perspective. Moreover, Frankl acknowledges many theorists, thinkers and researches throughout, giving each one of them their due credit to strengthen the value of his ideas.

The book addresses some serious concerns in two major sections—foundations of Logotherapy and applications of Logotherapy. In the truest sense, this is the book where his ideas of meaning and existential vacuum become more clear, applicable and introspective. While the preface gradually weaves the idea of the three basic pillars of freedom of will, will to meaning and meaning of life, the introduction section sets the stage of the theoretical, and practical understanding of the therapy. Through the means of a unique “I-thou encounter,” Frankl seems to reinforce Roger's core conditions of person-centered counseling. It is worthy to note how Frankl reiterates in his concepts and experiences, the very humanness of the unique human being.

There are several highlights of the book, such as the outright rejection of the reductionist approach, the idea of the two unique human capacities: self-transcendence and self-detachment, rejection of the “nothing-but-ness” view of human nature, the introduction of the dimensional ontology, the importance of values and conscience in finding meaning, the criticism of educational methodology today, the use of logic to emphasize on core concepts, etc. It is interesting to note how Frankl seems to entangle the human concepts with one another to explain his own point that a noological (existential) dimension must be considered to explain man. This is something that many practitioners also believe is the need of the hour. And the gradual weaving of concepts promotes his ability of logical reasoning, characterized by specification yet generalization, with a touch with oversimplification.

Clinicians and mental health practitioners would also excellently benefit from the explanation of the Logotherapeutic techniques—dereflection and paradoxical intention. With the help of case study reports and his own session dialogues with patients, Frankl explains how Logotherapy and its techniques can be employed for all types of neuroses (psychogenic, noogenic, and somatogenic) and disorders like schizophrenia and manic-depression. His dedication to explaining the techniques is noteworthy.

The peak moment for a reader bearing religious/spiritual beliefs comes in the conclusion section of the book where Frankl highlights the association between theology and Logotherapy graciously, with sufficient empirical evidences. Gradually from explaining the unconditional meaning in life, he introduces the presence of an ultimate being. Readers bearing faith in the writings of The Bhagavad Gita would agree in full compliance to this section, and find in Frankl's writings “all the arguments that he needs to strengthen his belief”, courtesy the similar presentation of ideas by Lord Krishna in the Gita and Frankl in his book (Shri Shrimad A. C. Bhaktivedanta Swami Srila Prabhupada, 1978).

Time and again Frankl also seems to stand on his words of re-humanizing the mental health services—including how doctors can choose to take a stand of their own neuroses and help their patients too, like has been done by Gadhia-Smith (2011).

However, for non-clinicians and impulsive readers, the ideas presented in this book may pose to be heavy to understand and may take a good while to not only comprehend but also absorb Frankl's ideas into their practice. Gradual and careful consideration of every line will benefit.

Numerous, extensive researches (quantitative and qualitative) have expanded Frankl's ideas and supported them; Frankl has mentioned quite a few of them in the book itself. In addition, Rainey (2014) had indicated that a vast majority of people crave for a sense of purpose while in the same year, Heintzelman and King (2014) suggested through summaries of epidemiological data and research that life is pretty meaningful and that meaning in life plays a role in adaptation. Several studies performed on a variety of population with various other variables have also reinforced Frankl's ideas. Loffler et al. (2012) have rightly proved that a stronger sense of meaning in life and a more elaborately structured personal meaning system correlated with a better mental health and more posttraumatic growth.

It would be appropriate to mention that the book is exhaustive yet very insightful to be summarized in a few words and that one aiming to prosper in life *per se*, would surely benefit from this book.

## Author contributions

The author is the sole contributor of this book review that encapsulates the careful consideration of the book in question.

### Conflict of interest statement

The author declares that the research was conducted in the absence of any commercial or financial relationships that could be construed as a potential conflict of interest.
